# Changing trends of *clostridium difficile* associated diarrhoea (cdad) in a tertiary care hospital in kolkata, india

**DOI:** 10.1186/2197-425X-3-S1-A118

**Published:** 2015-10-01

**Authors:** A Chakraborty, A Kar, S Roy

**Affiliations:** Infectious Disease, Medica Superspecialty Hospital, Kolkata, India; Critical Care, Medica Superspecialty Hospital, Kolkata, India; Microbiology, Medica Superspecialty Hospital, Kolkata, India

## Introduction

In this era of increased and indiscriminate antibiotic usage, it is not only the advent of multi-drug resistant pathogens, but also the rising prevalence of Clostridium difficile Associated Diarrhoea (CDAD) which is drawing concern world-wide. It is important not only to implement a comprehensive antibiotic policy, but also to take up stringent infection control measures to curb the rise of this serious infection.

## Objectives

Primary objective of this study was to know the prevalence and epidemiology of *Clostridium difficile* infection among the clinically suspected cases of Antibiotic associated diarrhoea in our hospital with a secondary objective of analysis of trend of CDAD over a period of 4 years.

## Methods

In this retrospective observational study we analyzed the prevalence and epidemiology of CDAD cases among all cases of clinically suspected antibiotic associated diarrhoea occurring in patients admitted in various departments of this hospital over a period of 4 years, since 2011 to 2014. The presence of *Clostridium difficile* in the stool samples was detected by ELISA for both toxin A and B. All infection control measures taken were recorded.

## Results

Out of 287 clinically suspected antibiotic associated diarrhoea cases reported from January 2011 to December 2014, Clostridium difficile toxin was detected in 57 (19.86%) occasions. The prevalence was on a rise after 2011 (10.52%), with a peak of 27% in 2013 and improved gradually over the next year (16.9% in 2014). Prior usage of broad spectrum antibiotics was among the prevalent risk factors identified.

## Conclusions

It was important to evaluate the prevalence of CDAD in this part of the country as there is increasing need of awareness among treating physicians on the risk factors and control of such infections. This study demonstrated the significant prevalence of *Clostridium difficile* Associated Diarrhoea (CDAD) in this hospital. Our study also depicted the importance of judiciously following antibiotic policies and strict infection control measures in a hospital. Furthermore it also raised the interest in future studies for the evaluation of community data on antibiotic usage and *Clostridium difficile* infection.
